# Harmonious Passion at Work: Personal Resource for Coping with the Negative Relationship between Burnout and Intrinsic Job Satisfaction in Service Employees

**DOI:** 10.3390/ijerph20021010

**Published:** 2023-01-05

**Authors:** Miriam Benitez, Alejandro Orgambídez, Francisco J. Cantero-Sánchez, Jose M. León-Pérez

**Affiliations:** 1 Department of Social Psychology, Faculty of Psychology, University of Seville, 41018 Seville, Spain; 2 Social Psychology Department, Faculty of Psychology, University of Málaga, 29071 Málaga, Spain

**Keywords:** work harmonious passion, burnout, intrinsic job satisfaction, service organizations

## Abstract

Research about harmonious passion as a personal resource that can have a protective effect in situations of stress and burnout is scarce but growing. Considering the job demands-resources (JD-R) model, the present study aims to address the above gaps by exploring the moderating role of harmonious passion at work in the relationship between burnout (physical fatigue, cognitive weariness, and emotional exhaustion) and intrinsic job satisfaction. The study sample consisted of 748 workers from service organizations (front-line employees) in southern Spain (Mage = 35.51, *SD* = 10.06, 52% women). Using statistical program R (R Core Team, 2022), the results of the regression models showed the moderating role of harmonious passion on the negative relationships between physical fatigue and intrinsic job satisfaction. In particular, at high levels of physical fatigue, employees with high scores on harmonious passion at work presented higher levels of intrinsic satisfaction compared with employees with low passion at work. That is, although service employees have high levels of physical fatigue, when they are passionate, they still possess satisfaction with their work. Therefore, our findings extend the JD-R theory by considering harmonious passion as a motivational resource that reduce feelings of burnout in service employees. Hence, it shows the importance of promoting the autonomous internalization of work, (through, i.e., job enrichment), which allows the development of harmonious passion at work and, therefore, increasing intrinsic job satisfaction.

## 1. Introduction

The current socio-economic context, characterized by the uncertainty of the job market and the difficulties of adapting to business expansion, is causing service employees to increasingly face hindrance demands from customers [1]. These situations are leading to the emergence of new elements and risk factors, especially psychosocial risks and those associated with chronic stress [2]. In this sense, the burnout syndrome emerges as one of the main psychosocial risks of service organizations [3,4], given its impact on the physical and psychological health of employees who have direct contact with the customers [5,6].

Burnout has become a popular way of describing the “personal agony” of work stress [7]. It is a subjective experience composed of negative cognitions, emotions, and attitudes toward work, peers, and one’s job role [8]. The effects of burnout range from depression and anxiety, psychological discomfort, or guilt, to physical pathologies such as cardiovascular diseases [3,4]. In this sense, burnout has recently been considered by the World Health Organization (WHO) as an occupational disease and it has been incorporated into the International Classification of Diseases (ICD). Given the recognition of the relevance of this syndrome in the workplace and its consequences for health, the importance of adapting preventive measures for its suppression or elimination in the most vulnerable employees is highlighted.

Faced with the traditional emphasis on the negative in occupational psychology—conflict or deteriorated interpersonal relationships, stress processes, work accidents, etc.—in recent years, a more positive view of occupational health has been consolidated, focusing on personal and job resources and their potential motivating effects [9]. The fundamental underpinning of this positive view of occupational health is, in the workplace, that not only is it important to prevent the negative consequences of psychosocial risk factors, but also to know how to promote the development of human talent in organizations, as well as enjoyment and well-being at the workplace [10]. The interest of this perspective is to apply the knowledge and experience of various disciplines to the safety, health, and well-being of employees. Among the various variables and strategies that are emerging in this regard, the concept of passion at work is worth underlining [11].

Passion at work can be defined as a strong orientation towards work activity, which is considered by employees as significant and in whose realization people dedicate time, effort, and both personal and organizational resources [12,13]. Two types of passion are distinguished: harmonious or autonomous and obsessive or compulsive. When passion is harmonious, work as an activity is internalized autonomously; that is, the person voluntarily “gets hooked” by deciding when and how to do it. If the passion is obsessive, the person is driven to work because they feel “forced” to do so, experiencing difficulties in disconnecting from matters related to work activity [12,13].

Several studies have related high levels of harmonious passion at work with higher levels of psychological well-being and positive emotions, such as enjoyment, job satisfaction, and organizational commitment [11,14,15]. However, few studies have focused on analyzing whether harmonious passion, as a personal resource, can have a protective effect in situations of burnout. Analyzing the buffer effect of harmonious passion is important for at least three reasons. First, it makes it possible to clarify the processes underlying the internalization of work and its possible protective effect in situations of stress. Second, it implies obtaining new evidence in favor of the role of personal resources in the process of health deterioration proposed by the JD-R model [9]. Finally, if our hypotheses are confirmed, this empirical evidence may allow for the development and implementation of interventions based on the enhancement of personal resources and, therefore, improving intrinsic job satisfaction, one of the main determinants of well-being and good performance in service employees. For all these reasons and following the JD-R theory [9], the main objective of the present study was to analyze the moderating role of harmonious passion at work in the relationship between burnout and intrinsic job satisfaction across a sample of service employees.

### 1.1. Burnout and Intrinsic Job Satisfaction

In recent years, burnout has been recognized as an important occupational psychosocial risk in service employees (tourism, hospitality, commerce) [1]. Employees who are permanently in contact with customers (front-line employees) are one of the groups most vulnerable to burnout, due to the emotional demands involved in successfully satisfying the customers. In this sense, front-line employees have to continually show emotional self-regulation to adapt to the customers’ needs and to offer a service of high quality. These characteristics of the workplace lead to feelings of burnout, especially if the employees are not highly motivated in their profession [16].

Since Freudenberger (1974) first detailed this syndrome as a “feeling of failure and an exhausting experience resulting from an overload of demands, personal resources, or spiritual strength of the worker” (p. 160), several definitions of burnout have been proposed [17]. In general, burnout can be considered a response to stress when functional coping strategies (cognitive and behavioral efforts) fail to handle specific external and/or internal demands, evaluated as overflowing the available resources [8]. Melamed et al. (1992), following the conservation of resources theory, define burnout as the experience of physical fatigue, emotional exhaustion, and cognitive weariness, which consume the energy and coping ability of the individual as a result of continuous and prolonged stress. Physical fatigue explains feelings of tiredness and low energy levels when performing daily work tasks. Cognitive weariness refers to the degree of deterioration and slowing of thought and mental agility at work. Finally, emotional exhaustion refers to the lack of energy to establish and/or maintain interpersonal relationships at work [18].

There is a strong consensus about the negative consequences of burnout on both personal and organizational results. Different reviews have shown relationships between burnout and more than a hundred symptoms that affect emotions and affects, cognitions, psychological well-being, attitudes, and behaviors at work [3,4]. The burnout syndrome has been related to anxiety and depression, difficulties concentrating at work, low levels of coping strategies, and low commitment and intention to stay [19,20]. In this sense, several studies have shown the negative consequences of burnout on job satisfaction [21,22]. The main explanation is that employees with high levels of burnout make more mistakes at work and are less thorough in their tasks, decreasing their performance. As a consequence, these negative responses at work affect the assessment of such work, translating into lower levels of both extrinsic and intrinsic satisfaction. Similarly, Lizano and Barak [23] conducted a three-wave longitudinal study with a sample of 361 workers in an urban public child welfare department in United States. They found that emotional exhaustion is negatively related to job satisfaction across all employees’ groups.

### 1.2. Passion at Work and Intrinsic Job Satisfaction

The concept of work passion has seen increased attention in the organizational behavior and management literature [24]. Passion can be defined as a strong orientation toward work, which is considered important and to which people dedicate time, effort, and resources, being able to distinguish between harmonious passion and obsessive passion [13].

Harmonious passion derives from autonomous internalization that occurs when individuals “have freely accepted the activity as important for them without any contingencies attached to it” [13] (p. 757). In this sense, harmonious passion is the result of the autonomous internalization of work into one’s identity; that is, a strong drive to voluntarily “be immersed” in the work. The person accepts that it is a relevant element of his/her identity, without being forced to think about it or to work all time. On the other hand, obsessive passion appears as the result of the forced and uncontrollable internalization of work into one’s identity, so that the employee feels a compulsive impulse to carry out the activity, even sacrificing time from other vital areas such as leisure or family [12,13].

Several studies have shown that harmonious passion increases work motivation and enhances psychological well-being and the meaning and significance of the tasks performed [11]. This is because such passion leads to positive emotional states during the performance of work. This voluntary way of engaging with work facilitates the person’s concentration, favoring experiences of absorption and improving their state of mind, leading them to experience higher levels of intrinsic job satisfaction [11,14,24].

### 1.3. The Moderating Role of Passion at Work

Harmonious passion has beneficial effects for both the individual and the organization. Employees who experience a healthy and autonomous work passion will show greater personal resources to perform tasks. In addition, harmonious passion has been shown to be negatively related to burnout and its effects. For example, it has been shown that harmonious passion can reduce the impact of emotional exhaustion on experienced psychological discomfort at work [11]. As a result, the possible moderating effect of harmonious passion could be considered similar to the effect of other variables, such as self-efficacy or optimism. Given that harmonious passion refers to a positive internalization of work and a liking for such work, this type of passion can be considered a personal resource that could buffer the negative effects of burnout on intrinsic job satisfaction [11]. For example, Lavigne and colleagues observed that higher levels of passion were related to the perception of greater resources to face tasks and work demands [14]. Similarly, Trépanier and colleagues found that harmonious passion increased the positive effect of personal resources on engagement and decreased the effect of job demands on burnout [15].

Based on this, harmonious passion could act as a moderator between burnout and intrinsic job satisfaction. That is, the impact of chronic stress on intrinsic job satisfaction will be less if the person feels passionate about the job. For example, receptionists who are passionate about their job will experience higher levels of intrinsic job satisfaction, even if they feel lacking in energy, stressed by the context or relationships with colleagues. Consequently, the following hypothesis is proposed:

**Hypothesis** **1:** *Harmonious passion will play a moderating role in the relationship between burnout and intrinsic job satisfaction, such that, in situations of high burnout (physical fatigue, cognitive weariness, and emotional exhaustion), employees with high scores on harmonious passion will perceive higher levels of intrinsic satisfaction, compared to people with low scores on harmonious passion.*

## 2. Materials and Methods

### 2.1. Study Design and Procedure

A correlational and cross-sectional design was carried out through convenience sampling, using questionnaires as an information collection technique. Before data collection, a power analysis was performed to determine the minimum size to detect the effects proposed in the study’s hypothesis. To do so, the Interaction PoweR package [25] was used, considering the following parameters: alpha of 0.05, power of 0.80, small moderation effect (*beta_int_* = 0.15), and reliability coefficients of the scales above of 0.80. Results from 10,000 simulations indicated a minimum of 512 participants.

Data collection was carried out through questionnaires applied individually and in person. The researchers contacted different companies that are usually involved in the academic activities of the university. After approval by the company’s board, the questionnaires were applied to all employees who voluntarily accepted to participate. Before beginning, participants provided their consent and the instructions for the questionnaires were explained. Confidentiality and anonymity of the data were guaranteed according to personal data protection regulations.

### 2.2. Participants and Inclusion Criteria

The sample was composed of 748 employees from service organizations (commerce, hospitality, and tourism) in southern Spain. Through convenience sampling, employees with a least one year performing the same job position were selected; any self-employed or unemployed persons were excluded. In total, 52% were women (*n* = 389), with a mean age for the sample of 35.1 (*SD* = 10.06), ranging between 18 and 61 years old. Concerning the type of job, the participants were classified using Hofstede’s proposal [26]: (a) 30.08% were unskilled or semi-skilled manual workers, (b) 22.06% generally trained office workers or secretary, (c) 17.91% were specialist technicians, (d) 19.25% were academically trained professionals or equivalent (but not a manager of people), and (e) 10.7% held positions as supervisors, managers, or administrators. The mean length of employment was 11.42 years (*SD* = 7.37) and 61.04% reported having a permanent contract.

No statistical differences were observed between men and women in the study variables: harmonious passion (t(734) = −1.516, *p* = 0.129), physical exhaustion (t(727) = 1.146, *p* = 0.252), cognitive weariness (t(735) = 1.341, *p* = 0.213), emotional exhaustion (t(736) = 1.106, *p* = 0.124), and intrinsic job satisfaction (t(716) = −0.263, *p* = 0.792). In relation to gender and type of work, the following significant differences were observed X2(4) = 26.47, *p* < 0.001): manual workers (57% men, 43% women, *p* = 0.03), secretary (38% men, 62% women, *p* < 0.001), specialist technicians (38% men, 62% women, *p* < 0.001), and academically trained professionals (40% men, 60% women, *p* = 0.03).

### 2.3. Instruments and Measures

To measure burnout, the Spanish version [27] of the Shirom-Melamed burnout measure (SMBM) [28] was used. The SMBM is composed of 14 items distributed in three dimensions: physical fatigue (6 items, e.g., “I feel tired”), cognitive weariness (5 items, e.g., “My way of thinking is slow”), and emotional exhaustion (3 items, e.g., “I feel like I’m not able to engage emotionally with my peers”). The response format used was Likert type, from never/not at all (1) to always/every day (7). The reliability, evaluated through the omega coefficient [28], was 0.93 (*boot se* = 0.005), 0.95 (*boot se* = 0.004), and 0.88 (*boot se* = 0.010) for physical fatigue, cognitive weariness, and emotional exhaustion, respectively.

Harmonious passion at work was measured using the Spanish version of Vallerand and colleagues’ scale, adapted by Orgambídez et al [12,13]. We used the 7 items related to harmonious passion (e.g., “This job allows me to live memorable experiences”). The items were answered using a Likert-type scale that ranged from totally disagree (1) to totally agree (7). The omega reliability coefficient obtained was 0.92 (*boot se* = 0.004).

Finally, intrinsic job satisfaction was measured with the 7 items related to the intrinsic dimension [29], of the overall job satisfaction questionnaire [30] (e.g., “The variety of tasks you perform in your job”). The items were answered using a Likert-type scale, from very dissatisfied (1) to very satisfied (7). The omega coefficient recorded in this study was 0.89 (*boot se* = 0.007).

## 3. Results

### 3.1. Preliminary Analyses

Before proceeding with the examination of the hypothesis, several tests were carried out to verify the possible effect of the common method variance (CMV) [31]. As all the data were based on self-report questionnaires and were collected in the same period, the common variance associated with the method may overestimate or underestimate the relationships between the variables. In this sense, it is recommended to carry out the Harman one-factor test to verify the possible influence of this bias.

All the items of the burnout, harmonious passion, and intrinsic job satisfaction scales were subjected to an exploratory factor analysis using the method of principal components with varimax rotation, forcing the extraction of a single factor. The existence of a variance problem associated with the common method would result in a factor with more than 40% of the variance extracted. The results of the exploratory factor analysis showed a factor that accounted for 34.8% so that, although the effect of the common method variance cannot be totally ruled out, it does not seem to significantly affect the relationships between the variables studied [31].

### 3.2. Descriptive Statistics and Correlations

Table 1 shows the mean, standard deviation, and correlations of the variables, as well as the confidence intervals of the omega reliability coefficients. Correlation analyses indicated that, as expected, all three burnout dimensions (physical fatigue, cognitive weariness, and emotional exhaustion) were negatively and significantly (*p* < 0.01) related to intrinsic job satisfaction: *r* = −0.19, *r* = −0.17, and *r* = −0.15 for physical fatigue, cognitive weariness, and emotional exhaustion, respectively. On the other hand, harmonious passion was positively related to intrinsic job satisfaction (*r* = 0.61, *p* < 0.01).

### 3.3. Moderation Analysis

To analyze the moderating role of harmonious passion in the relationship between burnout and intrinsic job satisfaction, several multiple linear regression models were carried out following the recommendations of Hayes [32]. The study variables were entered into the regression models in various steps. In Step 1, control variables were introduced (age, gender, and type of contract). In Step 2, the predictor (physical fatigue/cognitive weariness/emotional exhaustion) and moderator variables (harmonious passion) were entered. In Step 3, the interaction effect was added.

Hayes [32] recommends the use of bootstrap confidence intervals to verify the effect of moderation. In that sense, the lavaan package of the statistical program R [33,34] was used to calculate confidence intervals (bias-corrected confidence intervals), based on 10,000 samples and using the maximum likelihood method. If the confidence interval did not contain the zero value (0), the interaction could be considered significant. Before performing the regressions, the predictor and moderator variables were centered to reduce multicollinearity.

The results presented in Table 2 indicate the existence of a significant interaction: physical fatigue and harmonious passion on intrinsic satisfaction. We utilized the interaction package for plotting interactions, showing three conditional slopes of the predictor variable on the result variable: 1 *SD* above and 1 *SD* below the mean of the moderator variable. We also applied the Johnson-Neyman technique to probe interactions. This technique is recommended when the moderator variable is continuous and solves for the values of this variable for which the effect of the predictor variable in the results variable ceases to be significant.

**Figure 1:**
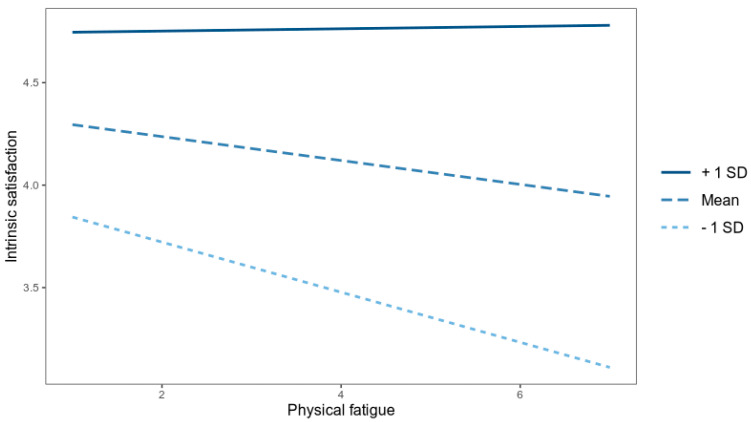
The relationship between physical fatigue and intrinsic job satisfaction by harmonious passion levels.

**Figure 2:**
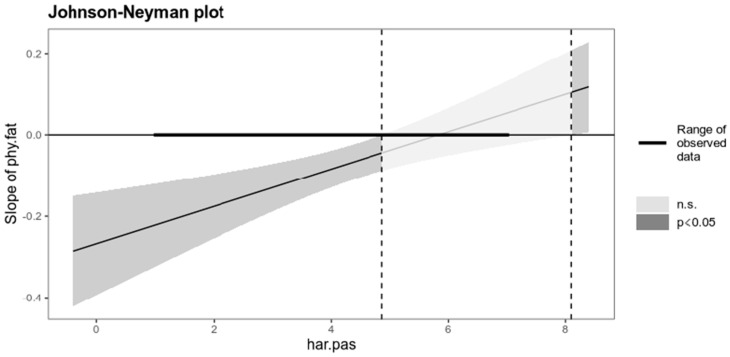
Johnson-Neyman graph for the interaction model.

Figure 1 shows the regression lines of physical fatigue on intrinsic job satisfaction for the mean ± 1 *SD*. According to the results of the Johnson-Neyman technique, physical fatigue ceased to be a significant predictor when the value of harmonious passion was equal to or greater than 4.87 (Figure 2).

## 4. Discussion

The impact of burnout on employees is such that it is considered one of the most important psychosocial risks in contemporary service organizations [35]. Several studies have associated burnout with physical and psychological pathologies [3,4,8]. From positive occupational health psychology, efforts are focused on interventions for the prevention of burnout and the enhancement of the strengths of workers. In this framework, we propose that harmonious passion at work can be considered a personal resource that could buffer the negative relationship between burnout and intrinsic job satisfaction.

Regarding the relationship between burnout and intrinsic job satisfaction, the results of the regression models showed that only physical fatigue was negatively related to intrinsic job satisfaction. Lack of energy, physical fatigue, and the feeling of “dead battery” decrease the levels of intrinsic satisfaction experienced by employees. However, cognitive weariness and emotional exhaustion do not seem to affect the intrinsic job satisfaction of the participants. These results seem contradictory to previous evidence that has shown a negative relationship between burnout and job satisfaction. For example, Amutio and colleagues observed, in a sample of healthcare professionals, that higher levels of burnout were related to lower levels of job satisfaction [21]. With a sample of nursing professionals, Figueireiro-Ferraz and colleagues also found burnout to be an important and significant predictor of job satisfaction over time [22]. A possible explanation is that the physical, cognitive, and emotional aspects of burnout may be differentially related to the intrinsic and extrinsic components of job satisfaction [36]. In other words, as intrinsic job satisfaction only considers the kind of work that is performed, a higher level of physical exhaustion because of your work is associated with a lesser satisfactory perception of your job. On the contrary, physical fatigue does not necessarily imply a negative perception of extrinsic aspects of job satisfaction, such as the pay or the relationship with coworkers and supervisors [27].

Regarding the role of work harmonious passion, as expected, it is significantly and positively related to intrinsic job satisfaction [24]. When work is internalized autonomously, when tasks at work are seen as “free” and “engaging” activities, passion can help generating positive emotional states during tasks performance. Consequently, people experience positive affective states and absorption in relation to work and its content. Being satisfied, defined as the degree of positive affect towards work and its components [22], the experiences caused by passion increase employees’ levels of satisfaction [13,15,24].

Furthermore, harmonious passion moderated the relationship between physical fatigue and intrinsic satisfaction. These results are in line with previous studies that have shown that pleasure, the emotion associated with passion for work, makes people withstand physical fatigue, even chronic fatigue, in a more positive way, and therefore increases levels of intrinsic satisfaction when passion is high [13,15]. With regard to the gender differences, no differences were observed between men and women in the levels of harmonious passion, burnout and intrinsic job satisfaction. The effects of passion on distress and well-being at work seem to be independent of the gender of the workers, although it would be necessary to consider the conditions of work performance of men and women in the companies.

Finally, this study has a series of elements that must be considered when interpreting the results. First, it is a cross-sectional design, which does not allow causal conclusions to be drawn about the relationship. Second, the survey design is especially sensitive to certain biases, such as the social desirability bias in the responses given or the trend in the responses of those people who voluntarily wanted to participate in the study. Moreover, all variables have been assessed using self-reports, which increases the risk of variance due to the common method (CMV). Despite that the Harman test indicates that CMV does not seem to be an element that distorts the observed relationships, further research may incorporate other sources of information and longitudinal designs. In addition, future studies should focus on the identification of the psychological processes underlying harmonious passion, both in its mediating role between job resources-demands and engagement-burnout, and in its moderating role between burnout and job satisfaction, psychological well-being, and physical health. Personal variables, such as optimism or self-efficacy, and organizational variables, such as organizational justice, may clarify the processes involved, as well as studies with different professional groups.

## 5. Conclusions

Harmonious passion appears to work as a moderating variable in the relationship between burnout and intrinsic job satisfaction. In addition to being considered a personal resource that would allow coping with the demands of the work context, it reduces the effects of chronic stress, especially those related to physical fatigue and satisfaction. Considering that harmonious passion is derived from an autonomous internalization of work, organizations can carry out interventions based on the enrichment of work content, designing jobs with meaning, a variety of tasks, and jobs which are balanced in terms of job demands-resources [9]. These interventions would favor the development of a harmonious passion for work while increasing levels of intrinsic satisfaction, even in situations of high stress.

## Figures and Tables

**Table 1:** Descriptive statistics and correlations (*n* = 748).

	1	2	3	4	5
1. Physical fatigue	(0.92, 0.94)				
2. Cognitive weariness	0.71	(0.94, 0.96)			
3. Emotional exhaustion	0.48	0.67	(0.86, 0.90)		
4. Harmonious passion	−0.19	−0.17	−0.15	(0.87, 0.90)	
5. Intrinsic job satisfaction	−0.20	−0.14	−0.14	0.61	(0.91, 0.93)
Mean	2.98	2.61	2.27	4.57	4.16
Standard deviation	1.29	1.27	1.32	1.39	0.99

Note: All coefficients are statistically significant (*p* < 0.01). The confidence intervals of the reliability coefficients of the scales on the diagonal

**Table 2:** Analysis of the moderating role of harmonious passion in the relationship between burnout and intrinsic job satisfaction (*n* = 748).

	Est (B)	BC 95% CI		t Value	*p* Value
		2.5%	97.5%		
(Intercept)	4.275	4.184	4.365	92.669	0.000
Sex	0.016	−0.094	0.126	0.284	0.777
Age	−0.002	−0.007	0.003	−0.739	0.460
Type of contract	−0.298	−0.422	−0.175	−4.761	0.000
Physical fatigue	−0.060	−0.103	−0.017	−2.730	0.006
Harmonious passion	0.418	0.378	0.458	20.397	0.000
Interaction	0.039	0.013	0.066	2.954	0.003
	*F*(6,743) = 85.745, *p* < 0.01, Ad. *R*^2^ = 0.404				
(Intercept)	4.266	4.174	4.358	91.132	0.000
Sex	0.004	−0.107	0.116	0.074	0.941
Age	−0.002	−0.008	0.003	−0.850	0.396
Type of contract	−0.303	−0.428	−0.178	−4.770	0.000
Cognitive weariness	−0.027	−0.072	0.017	−1.217	0.224
Harmonious passion	0.425	0.385	0.466	20.591	0.000
Interaction	0.015	−0.014	0.043	1.005	0.315
	*F*(6,738) = 79.988, *p* < 0.01. Ad. *R*^2^ = 0.389				
(Intercept)	4.285	4.194	4.376	92.591	0.000
Sex	−0.006	−0.116	0.105	−0.098	0.922
Age	−0.003	−0.008	0.003	−0.990	0.322
Type of contract	−0.306	−0.430	−0.183	−4.869	0.000
Emotional exhaustion	−0.034	−0.077	0.009	−1.564	0.118
Harmonious passion	0.427	0.397	0.467	20.916	0.000
Interaction	0.027	−0.001	0.055	1.890	0.059
	*F*(6,746) = 83.345, *p* < 0.01, Ad. *R*^2^ = 0.397				

Note: BC—Bias Corrected confidence intervals based on 10,000 samples.

## Data Availability

The data presented in this study are available on request from the corresponding author. The data are not publicly available due to confidential agreements with the companies involved in the study.
